# RUNDC3A/SNAP25/Akt signaling mediates tumor progression and chemoresistance in gastric neuroendocrine carcinoma

**DOI:** 10.1038/s41419-022-05294-7

**Published:** 2022-10-01

**Authors:** Ziqi Lin, Hang Fai Kwok

**Affiliations:** 1 Cancer Centre, Faculty of Health Sciences, University of Macau, Avenida da Universidade, Taipa, Macau SAR; 2 MoE Frontiers Science Center for Precision Oncology, University of Macau, Avenida de Universidade, Taipa, Macau SAR; 3 Department of Biomedical Sciences, Faculty of Health Sciences, University of Macau, Avenida de Universidade, Taipa, Macau SAR

**Keywords:** Prognostic markers, Neuroendocrine cancer

## Abstract

Gastric neuroendocrine carcinoma (GNEC), a heterogeneous group of neuroendocrine neoplasms (NENs) derived from gastric neuroendocrine cells, has been shown to be more aggressive and chemoresistant in gastric cancer, which contributes to the poor prognosis. We analysed transcriptome profiles of tumor/non-tumor tissue from GNEC patients and GNEC cell lines to explore the underlying mechanisms. Our results suggest a critical role for synaptosomal-associated protein 25 kDa (SNAP25) in GNEC. SNAP25 was found to stabilize Akt via modulating its monoubiquitination. We further identified RUN domain containing 3A (RUNDC3A) as an upstream molecule that regulates SNAP25 expression, which is associated with tumor progression and chemoresistance in GNECs. Moreover, these findings were extended into multiple NENs including neuroendocrine carcinomas in the intestinal tract, lungs and pancreas. Identifying the RUNDC3A/SNAP25/Akt axis in NENs may provide a novel insight into the potential therapeutic target for patients with NENs.

Neuroendocrine carcinomas (NECs) are currently described as poorly-differentiated neuroendocrine neoplasms (NENs) derived from the diffuse endocrine system and distributed throughout the human body [1]. Accounting for ~4% of all neuroendocrine tumors, gastric neuroendocrine carcinoma (GNEC), a subtype of gastroenteropancreatic neuroendocrine tumors (GEP-NETs), arising from enterochromaffin-like (ECL) cells, is a rare malignant cancer found in the stomach, presenting various characteristics differences from gastric carcinomas (GCs) [2, 3]. With increasing incidence in many countries, GNEC patients had a poorer prognosis than GC patients due to their high malignancy and irresponsiveness to traditional chemotherapy [3]. Therefore, it is crucial and significant to improve the therapeutic effect by identifying the molecular machinery underlying tumor growth and drug resistance in GNEC.

As elucidated in our previous work, we analysed transcriptome profiles of tumor/non-tumor tissue from GNEC patients and GNEC cell lines. A panel of key genes was identified specifically dysregulated in GNEC [4]. Gene Ontology (GO) and pathway enrichment analysis preliminarily revealed a close relationship between synapse-related biological functions and GNEC, and SNAP25 was remarkably involved in these processes [5]. Synaptosomal-associated protein 25 (SNAP25) is an indispensable component of the soluble N-ethylmaleimide-sensitive factor attachment protein receptor (SNARE) complexes which regulate membrane fusion during endocytosis of synaptic vesicle, mostly expressed in neuron-like cells [6]. SNAP25 was found essential in lysosomal trafficking processes and involved in the autophagy pathway, which could subsequently promote SNAP25-expressing epithelial cancers [7]. The expression level of SNAP25 in diffuse large B-cell lymphoma was correlated with patient survival and prognosis [8]. Although SNAP25 proteins are also abundant in ECL cells [9], whether characteristics of malignant findings and multidrug resistance in GNEC implicate SNAP25 remains unknown, nor has its mechanism been thoroughly explored.

To investigate the role of SNAP25 in GNEC, our recent study was conducted and published in *Cell Death Discovery* [5]. From in vitro and in vivo studies, we observed that SNAP25 knockdown reduced GNEC cells proliferation and tumor growth, as well as increased susceptibility of GNEC cells to multiple chemotherapeutic agents 5-fluorouracil and paclitaxel. Next, we investigated the mechanism by which SNAP25 regulates GNEC. Protein expression analysis showed that the absence of SNAP25 caused a decrease in total Akt protein level and the same reduction in Akt activation (Ser-473). Further results from RT-PCR demonstrated that SNAP25 did not affect Akt mRNA status. While using cycloheximide (CHX) to inhibit protein synthesis, immunoblotting illustrated a longer Akt protein half-life in GNEC cells expressing SNAP25. In addition, in vitro ubiquitination analysis of Akt displayed an elevated monoubiquitination level in GNEC cells after SNAP25 knockdown. Interestingly, we detected a SNAP25-Akt interaction by performing co-immunoprecipitation. These results suggested that SNAP25 might interact with Akt and modulate its monoubiquitination, which could prevent Akt protein from proteasome-dependent degradation in GNEC (Fig. 1).

**Fig. 1:**
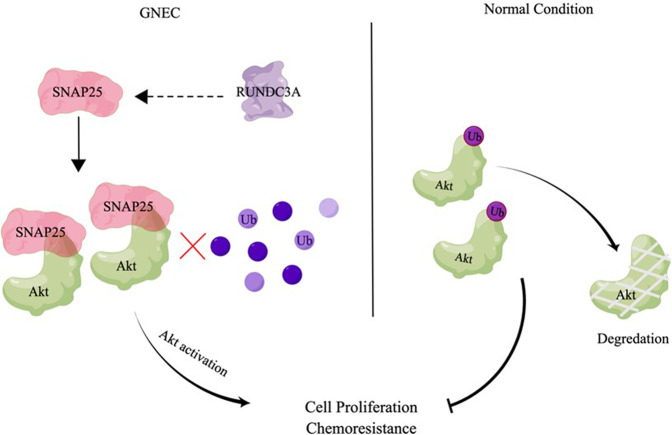
Schematic representation for the mechanism of RUN Domain Containing 3A (RUNDC3A)/Synaptosomal-Associated Protein 25 kDa (SNAP25)/Akt axis. The mechanism of RUNDC3A/SNAP25/Akt axis serves as a switch that regulates Akt activation in gastric neuroendocrine carcinoma (GNEC) progression and chemoresistance by stabilizing cytoplasmic Akt. The curve arrows shown in the diagram indicate as positive effect, and the curve T-shaped line indicates as inhibitory effect. See text for further details, as well as the original study by Chen and colleagues [5].

In addition to the stomach, neuroendocrine cells are widely distributed throughout the human body, including the intestinal tract, lungs, and pancreas [10, 11]. To extend our observations to NENs, a comparative analysis of expression profiles was performed among GNEC and multiple NENs (PNET, SCLC, and SINEC), and a similar panel of synapse-related pathway enrichment was observed. Meanwhile, a protein–protein interaction network analysis presented SNAP25 in the center of other differentially expressed genes, which account for synapsis-related cellular functions. These findings are consistent with early reports that neurogliomal synapse-related pathways dysregulation occurs in both primary and metastatic glioma, and their enhancement drives brain tumors progression and induces chemoresistance [12, 13]. Therefore, our data indicated that SNAP25 might play a critical role in synapsis-related cellular functions and the growth of multiple NENs.

According to database analysis, it is also worth noting that RUNDC3A appears to be another common differential expressing genes in NENs. Indeed, we validated that genetic depletion of RUNDC3A significantly reduced GNEC tumor growth both in vitro and in vivo. Given their similar effect on tumor progression, RUNDC3A expression positively correlated with SNAP25 expression in GNEC patients. In addition, a total of lung, pancreas and small intestine NEN datasets were enrolled for similar correlation analysis, and we verified a significant correlation between RUNDC3A and SNAP25 expression in these cohorts. Further proof of western blot identified SNAP25 protein levels were reduced in RUNDC3A knockdown GNEC cells, while regulation of SNAP25 didn’t affect RUNDC3A protein abundance. Taken together, these results suggested RUNDC3A is a novel regulator in the upstream of SNAP25-mediated GNEC progression, which might also potentially involve NENs development.

Collectively, our study demonstrated a novel mechanism in which overexpression of RUNDC3A and SNAP25 modulates Akt protein stability to enhance tumor growth and chemoresistance in GNEC. Given the common regulation of SNAP25 and the association between RUNDC3A and SNAP25 in multiple NENs, we conjectured that such mechanism described above is of general applicability. Additionally, considerable research has indicated that PI3K/Akt/mTOR pathway plays a major oncogenic role in many cancer types including neuroendocine carcinoma [14, 15], which conforms to our observation of activation of Akt in Ser-473 residue. And it is therefore reasonable to speculate that modulation of Akt activity by RUNDC3A/SNAP25 may have crosstalk with other Akt-related pathways. Further studies are required to verify our hypothesis. From a therapeutic perspective, existing therapy directly targeting Akt and other major components of the pathway has emerged as pharmaceutical imperfection. Our observation of RUNDC3A/SNAP25/Akt axis in NENs progression may provide a novel insight into the potential therapeutic target for patients with NENs.
